# Editorial: Epidemiological studies on Japanese diets, health, and nutritional outcomes

**DOI:** 10.3389/fnut.2024.1448258

**Published:** 2024-07-03

**Authors:** Akio Shimizu, Yasutake Tomata

**Affiliations:** ^1^Department of Rehabilitation Medicine, Mie University Graduate School of Medicine, Tsu, Japan; ^2^School of Nutrition and Dietetics, Faculty of Health and Social Services, Kanagawa University of Human Services, Yokosuka, Japan

**Keywords:** diet, healthy aging, nutrients, mortality, life expectancy

Since the 1970s, life expectancy in Japan has been among the highest in the industrialized world. It is also characterized by a low ischemic heart disease mortality rate and a low prevalence of obesity compared to Western countries. It is hypothesized that these characteristics have been influenced by the unique dietary habits of the Japanese people over the past half-century. Therefore, further evidence on the dietary habits of the Japanese may provide valuable insight into healthy eating habits. Based on a previous study, typical components of the Japanese dietary pattern are defined by a higher intake of rice, miso soup, seaweed, pickles, green vegetables, seafood, and green tea, and a lower intake of red meat (1). Although some of these components overlap with the Mediterranean dietary pattern, there are fewer epidemiological studies on the Japanese dietary pattern compared to the Mediterranean dietary pattern.

This Research Topic covers papers on the impact of the Japanese dietary pattern on health and nutritional outcomes. There are four papers on this Research Topic covering the above aspects.

The Japanese dietary pattern is known to contribute to health status by providing many beneficial nutrients. Domoto et al. reported that taurine, one of the nutrients found in seaweed and seafood, is inversely correlated with lower extremity muscle strength. Previous studies have reported that adherence to the Japanese dietary pattern is protective against declining muscle strength (2) and mobility capacity (3) in the older population. According to Domoto et al., adherence to the Japanese dietary pattern preserves muscle health, which may be related to a high intake of taurine. Kawamura et al. reported that the Japanese dietary pattern may have protective effects against biological aging. Epidemiological studies on the Japanese dietary pattern to date have reported protective effects on mortality and disability incidence (1). These mechanisms have been considered to be related to the fact that the Japanese dietary pattern contains a high proportion of nutritious foods that are beneficial to health. However, there is little biological evidence of the effect of the Japanese dietary pattern on health extension. According to the findings of Kawamura et al., Japanese men who adhere to Japanese dietary patterns are positively correlated with age-adjusted DNAm-based telomere length. These findings suggest that the Japanese dietary pattern may be more beneficial to the human body from a micro perspective. Taking these reports into account, Japanese dietary pattern adherence may contribute to healthy life expectancy at the cellular level through beneficial nutrient intake.

In considering the application of the Japanese dietary pattern, it is also important to consider the shortcomings of the components. Nanri et al. reported that a high intake of green tea, a component of the Japanese dietary pattern, was inversely correlated with serum ferritin concentrations. Green tea is known to contain catechins, which are antioxidants, but it also contains high levels of tannins, which inhibit iron absorption. This study suggests the need for moderate green tea consumption in adherence to Japanese dietary patterns. Furthermore, adherence to the Japanese dietary pattern results in high sodium intake. Increased salt intake increases the risk of cardiovascular disease via increased blood pressure. In a randomized controlled trial, Sasaki et al. demonstrated that a milk-based culture of white mold improved cognitive function and sleep quality. White mold is used to produce Camembert cheese. Therefore, incorporating dairy products into the traditional Japanese diet may maximize the benefits to health status. Future epidemiological or *in vitro* validation of healthier modern Japanese dietary patterns is needed.

In summary, this Research Topic includes nutrients and mechanisms that support current evidence that the Japanese dietary pattern improves healthy life expectancy. It also suggests the need for future research on the components of the Japanese dietary pattern that are more beneficial to health.

## Author contributions

AS: Writing – original draft, Writing – review & editing. YT: Writing – original draft, Writing – review & editing.
